# Fishmeal replacement by periphyton reduces the fish in fish out ratio and alimentation cost in gilthead sea bream *Sparus aurata*

**DOI:** 10.1038/s41598-021-00466-5

**Published:** 2021-10-25

**Authors:** Gilda Savonitto, Roy Barkan, Sheenan Harpaz, Amir Neori, Helena Chernova, Antonio Terlizzi, Lior Guttman

**Affiliations:** 1 Israel Oceanographic and Limnological Research, The National Center for Mariculture, 8811201 Eilat, Israel; 2 Department of Life Sciences, University of Trieste, Trieste, Italy; 3 Department of Life Sciences, Ben-Gurion University of the Negev Eilat Campus, 84105 Beer-Sheva, Israel; 4 Department of Poultry and Aquaculture, Institute of Animal Science, Agricultural Research Organization, Rishon LeZion, Israel; 5 Morris Kahn Marine Research Station, Marine Biology Department, The Leon H. Charney School of Marine Sciences, University of Haifa, 3498838 Haifa, Israel; 6 The Interuniversity Institute for Marine Sciences in Eilat, 8810302 Eilat, Israel; 7 Stazione Zoologica Anton Dohrn, Napoli, Italy

**Keywords:** Environmental impact, Ichthyology

## Abstract

Aquaculture threatens natural resources by fishing down the sea to supply fishmeal. Alternative protein sources in aquafeeds can provide a solution, particularly those that are waste from other operations and thereby reduce feed production costs. Toward this goal, we examined the waste biomass of marine periphyton from biofilters of an integrated multi-trophic aquaculture (IMTA) system as a replacement for fishmeal in diets of gilthead seabream (*Sparus aurata*). Four isoproteic (41%) and isolipidic (16.7%) aquafeeds were formulated with increased content of periphyton and a corresponding decrease in fishmeal from 20 to 15, 10, or 0%. The growth and biochemical content of seabream fingerlings (initial body weight 10 g) were examined over 132 days. Replacing 50% of fishmeal by waste periphyton improved feed conversion ratio (1.2 vs. 1.35 in the control diet) without harming fish growth. The complete replacement of fishmeal with periphyton resulted in 15% slower growth but significantly higher protein content in the fish flesh (59 vs. 52% in the control diet). Halving fishmeal content reduced feed cost by US$ 0.13 kg^−1^ feed and saved 30% in the cost of conversion of feed to fish biomass (US$ 0.58 kg^−1^ produced fish vs. $0.83 in the control diet). Finally, the total replacement of fishmeal by waste periphyton in the diet reduced the fish in—fish out ratio to below 1 (0.5–0.9) as compared to 1.36 in the control diet. Replacing fishmeal with on-farm produced periphyton minimizes aquaculture footprint through the removal of excess nutrients in effluents and the use of waste biomass to reduce the ‘fish in’ content in aquafeeds and fish production costs. The present study demonstrates the great practical potential of this dual use of marine periphyton in enhancing the circular economy concept in sustainable fish production.

## Introduction

The rapid expansion of the aquaculture industry in recent decades aims at closing future food gaps through the supply of quality protein. However, maintaining its sustainability is a primary goal, with much attention devoted to the improvement of farms’ footprint, environmental friendliness and cost-effectiveness. One example is commercial aquafeed for carnivorous marine fish which is very high in protein content, up to 50% of pellet dry biomass, but much of that is in the form of fishmeal^1^. Although the cost of fishmeal has been reduced in recent years to around 1500 USD per tonne, with the limitation in natural resources for this ingredient due to overfishing^2,3^ it is expected to increase in the future. Currently, about 90% of produced fishmeal derives from small pelagic fish species as anchovies, sardines, mackerel, capelin, and menhaden^4^ while there is continuing concern over the impact of fishing pressure on predator–prey relationships in the stressed marine ecosystems. Moreover, the high content of digestive enzymes in these species leads to post-harvest solubilization and difficulties in handling and processing, with rapid losses of both extracted protein and oil^5^. Following the predicted scarcity of fish and greater attention to circular economy in the seafood sector, much effort has been made to reduce fishmeal content in aquafeeds by the use of alternative protein from land crops such as soy, corn and wheat^2,6^. However, the use of such alternatives in diet may result in poorer growth performance of fish, as reported in sea bream^7–9^ and Atlantic salmon^10,11^. This is probably due to the presence of anti-nutritional factors in some of these raw materials which harm nutrient digestion and absorption^12,13^. However, another constraint to increasing plant meal and oil content in aquafeeds is the consequent allocation of more land and water required to produce such plants. This can undermine the sustainability of these alternative protein sources. Hence more focus should be given to protein-rich and nutritious agricultural waste products, particularly ones that are derived from aquaculture and may be efficient for reduction of this industry’s footprint by their further use in fish nutrition. This should also allow reduction of the ‘fish in’ levels in aquaculture and the minimizing of production cost for both aquafeeds and fish.

In recent years macroalgae such as the green seaweed *Ulva* sp. (Chlorophyta) have been evaluated for their potential as alternative protein to replace fishmeal in the diets of various fish including carnivorous marine species such as seabass and seabream^14–16^. However, in many cases protein content in *Ulva* was far poorer than that in fishmeal or formulated feed^17–19^ and required boosting by additional resources such as poultry meal or soy protein extract. Integrated multi-trophic aquaculture technology (IMTA) appears to have a positive effect on macroalgae protein content as a result of its culture in nutrient-rich fishpond effluent^20,21^. In fact, when cultured in fishpond effluent, *Ulva* sp. assimilated much of the available ammonia–nitrogen into protein-rich biomass, with up to 37% protein in dry biomass^15,22^. Such biomass led to successful results in nutrition of various organisms such as sea urchins^23,24^, molluscs^25^, and fish^15,26^.

However, the fact that fishpond effluent contains nitrate-nitrogen is a major drawback in the use of macroalgae biofilters, as uptake of this N-form by the algae requires great energy investment compared to uptake of ammonia. In recent years, marine periphyton has been identified for its potential in bio-conservation of waste nutrients in fishpond effluent, showing a simultaneous removal of both N-forms: ammonia and nitrate^22,27^. This is due to the co-existence of various organisms such as micro- and macro-algae, bacteria and other microorganisms in the periphyton mat^28,29^. Moreover, in contrast to the high aeration required in *Ulva* biofilters, marine periphyton can be cultured relatively cheaply in unaerated biofilters requiring only light and artificial substrate like plastic nets for development ^30^, while the oxygen produced in the biofilters can be further used to support fish production in the primary culture ponds ^27,31^. As in other plant/algal biofilters, frequent harvesting of the periphytic biomass is required to keep both biomass production and nutrient removal rapid^22,30^, while the protein-rich harvested biomass is considered waste unless used for other purposes.

Nutrition-wise, periphyton has a long history of supporting fish production worldwide, as seen in culture ponds of freshwater fish like tilapia^32–34^, carp^35,36^, mosquitofish^37^ and shrimps^33,38,39^ and in brackish-water culture ponds for mullets^40^ and milkfish^41^. In some cases, application of periphyton technology in culture ponds resulted in as much as a 40% saving in commercial aquafeed^42^. However, it is still questionable whether the technology is limited to extensive culture ponds of the hitherto reported herbivorous and omnivorous species versus fish of a higher trophic level. One attractive candidate may be the marine gilthead sea bream (*Sparus aurata*, Sparidae) that has a wide distribution in various aquaculture settings including off-shore cages, and land- based IMTAs and recirculating aquaculture systems (RAS). This has resulted in an adequate amount of data on the growth of this species under various conditions^43^ to allow comparison of performance with respect to feeding regime or dietary ingredients. Toward this goal, the current study examined marine periphyton from an IMTA biofilter as a replacement for fishmeal in the diet of gilthead seabream as a model carnivorous marine fish.

## Results

### Fish growth performance

Weight gain during the feeding trial was relatively similar under the three diets containing fishmeal (*P* = 0.4), ranging between 113 and 136 g, even when the amount of this ingredient was half that of the control commercial feed (Table 1). Under these diets, fish growth was rapid and relatively similar, with SGR between 1.9 and 2% BW day^−1^ (*P* = 0.5). Compared to the other diets, feeding with the fishmeal-free diet resulted in slower growth of 1.67% BW day^−1^ (*P* < 0.05). However, a significant deceleration in growth under this diet started only somewhere between days 72 and 93 of the experiment, as until then fish weight on this diet was similar to that of any other diet (*P* > 0.05). During the first 72 days fish SGR in all diets varied between 1.7 and 1.9% BW day^−1^ and began to decrease in the fishmeal-free diet only afterwards, until reaching SGR of 0.65 between days 117 and 132 (or only 1% BW day^−1^ between days 72–132; not shown). Increase in fish biomass as body weight correlated with that of gained length in all diets and the calculated ratio between these parameters (weight:length) was 1.14 ± 0.008, 2.13 ± 0.03, and 6.2 ± 0.11 (mean ± SE) on days 0, 32, and 132, respectively. However, above a weight of ~ 150 g, fish length remained relatively constant (~ 23 cm; not shown). The RGL ratio of gut:body length was similar under different diet regimes (*P* = 0.8), between 0.85 and 0.96 (Table 1). Similarity between different diets was also measured in terms of the ILW index, which ranged between 1.4 and 4.3 (Table 1). Survival rate was also relatively similar under all diets (*P* = 0.7), ranging between 70 and 78% (Table 1).

**Table 1:** Fish performance under different diet regimes with periphyton and reduced fishmeal content as compared to the periphyton-free control diet with fishmeal.

	Control	Low periphyton	High periphyton	High periphyton–no fishmeal
Initial length (cm)	8.9 ± 0.1	8.9 ± 0.1	8.8 ± 0.1	8.9 ± 0.1
Final length (cm)	19.9 ± 0.5	20.3 ± 0.4	20.0 ± 0.2	18.8 ± 0.4
Initial weight (g)	9.95 ± 0.2	10.2 ± 0.2	10 ± 0.2	10.3 ± 0.2
Final weight (g)	133.6 ± 6	140.1 ± 6	128.3 ± 6	93.8 ± 4
Gained biomass (g)	123.6 ± 2.8	129.8 ± 3.6	118 ± 2.6	84 ± 4.8
RGL	0.8 ± 0.11	0.8 ± 0.07	0.9 ± 0.02	0.9 ± 0.1
ILW	2.91 ± 0.41	3.29 ± 0.29	3.38 ± 0.08	3.5 ± 0.39
SGR (% d^−1^)	1.97 ± 0.03	1.98 ± 0.04	1.93 ± 0.03	1.67 ± 0.04
FCR	1.35 ± 0.06	1.17 ± 0.03	1.22 ± 0.04	1.47 ± 0.11
Survival (%)	72	78	70	74
PER	1.83 ± 0.1	2.03 ± 0.05	1.94 ± 0.07	1.69 ± 0.13
PPV	33.7 ± 1.8	39.7 ± 1.7	40.7 ± 4.2	33.8 ± 3.6

All values are mean ± SE. n = 90. SGR, FCR, survival, PER, and PPV were calculated following mean values for each replicate of tanks, n = 3.

The rate of feed conversion to biomass (FCR) was relatively similar under the different diets (*P* = 0.07) and ranged between 1.17 and 1.47 (± 0.05) (Table 1). While the lowest efficiency in feed conversion was measured in the fishmeal-free diet, other diets with periphyton (12.5 or 25% periphyton and reduced fishmeal) resulted in the highest FCR.

### Fish biochemical content and protein conversion to fish biomass

Fish fed different diets revealed no significant differences in their biochemical content of lipids, carbohydrates, or ash (*P* < 0.01; Table 2). However, trends of reduction in lipid content with the reduction in fishmeal content were seen between different diet types, while corresponding trends of a respective increase in both the ash and protein content were also observed (Table 2). Fish fed on the high periphyton/fishmeal-free diet gained significantly more protein in their dry body biomass (59%) as compared to their counterparts fed the fishmeal-rich control diet, where protein content in dry biomass was only 52% (*P* = 0.14; Table 2). No differences were measured between fish under different diet types in terms of the PER or PPV (*P* > 0.1).

**Table 2:** Biochemical content in fish fed with different diets.

	Control	Low periphyton	High periphyton	High periphyton–no fishmeal
DW	36.4 ± 1.5	35.6 ± 1.4	36.3 ± 1.8	36.2 ± 3.8
Protein	51.7 ± 1.6	52.6 ± 1.8	55.8 ± 1.6	58.8 ± 2.4
Lipids	34.2 ± 3.2	33.4 ± 2.9	25.6 ± 3.3	22.9 ± 4
Ash	13.7 ± 1.4	13.2 ± 1.5	16 ± 1.5	15.2 ± 1.5

All values with the exception of DW are % of dry weight. Values are mean ± SE, n = 9.

## Discussion

Marine periphyton from an IMTA biofilter system plays a valuable role in the removal of excess nitrogen and phosphorus from fishpond effluent and assimilating them into edible biomass for further use^44–46^. This is in addition to its other advantages in water oxygenation and pH balancing^29,47^. In earlier studies, periphyton in culture ponds reduced the amount of commercial aquafeeds required^36,42,46^ but the potential of this edible biomass in replacing dietary fishmeal has not been reported until now. Therefore, the current study is novel in its evaluation of the feasibility of having marine periphyton from a biofilter for fishpond effluent treatment also serve as a protein source to minimize fishmeal content in pelleted diets and enhance the circular economy concept. In the present study, milled periphyton enabled reduction of the overall animal ingredients in the feed of *S. aurata* from 42 to 32 or even 22% by minimizing fishmeal content by 50 or 100%, respectively. Although a complete removal of fishmeal from feed resulted in 15% slower growth, halving fishmeal content in the feed was highly successful in terms of fish growth performance and feed conversion to biomass. In fact, the SGR and FCR measured here in the halved fishmeal content treatment are comparable with, and in some cases even surpassed, the measured ratios in previous studies of sea bream on high-fishmeal diets^7,8,15^. While these results are promising, the fish survival rate of between 70 and 78% could be considered relatively low. However, fish mortality occurred regardless of diet type (including control) or culture tank and mainly transpired a few days after fish weighing, perhaps due to the stress of recurring anaesthesia. Another explanation may be the relatively long duration of the current experiment, as the mortality rate of 22–30% measured over 4.5 months of culture is comparable to that seen in previous studies on *S. aurata* where fish mortality rate reached 40^8^, or 39^48^, or 23%^49^ during culture periods of 5, 7, or 7.5 months, respectively.

Economically, fishmeal reduction with either the lower or higher amount of periphyton maintained fish performance while reducing feed cost by 11 or 21%, respectively, and feed cost of the fishmeal-free diet was only 65% that of the control aquafeed (Table 3). Moreover, when considering the amount of feed required to gain the resulting fish biomass under each examined diet type, the saving in feed cost for each 1 kg of fish produced during the entire 132 days of experiment was calculated at 22% or 30% when providing diets with low or high periphyton (w./w.o. fishmeal), respectively (Table 3). The saving in feed cost by the use of periphyton in seabream diet in the present study is consistent with an earlier report on the replacement of fishmeal with the green seaweed *Ulva lactuca* in which a high ratio of *Ulva* in feed saved US$ 0.25 for each kg of produced feed^15^.

**Table 3:** Economic calculations of feed costs (in US $ kg^−1^ feed) and fish production (in US $ kg^−1^ produced fish) in current study.

Costs	Control	Low periphyton	High periphyton	High periphyton–no fishmeal
Feed (US $ kg^−1^ feed)	0.63	0.58	0.5	0.41
Fish production (US $ kg^−1^ produced fish)	0.83	0.58	0.64	0.59

Data for calculation of feed cost came from local suppliers of feed ingredients or the available price in the Index Mundi database on the day of preparation. The cost of fish production was calculated for each diet type based on the cost of feed and the calculated feed to biomass conversion ratio (FCR) in the current study during 132 days of culture.

The reduction in fishmeal content in feed benefits both the environment and the aquaculture industry by reducing the amount of wild caught fish required for supply of this ingredient in fish diet. Data from the marine ingredients organisation (IFFO) estimated fishmeal yield from wild-caught fish at 22.5%^50,51^, suggesting that using this study’s control diet, 1111 kg of wild-caught fish would be needed to supply the fishmeal required for production of 1000 kg fish (1250 kg feed × 0.2 fishmeal content in feed/0.225 fishmeal yield). Using periphyton in a diet and only 10% fishmeal, this amount of wild-caught fish can be nearly halved to only 578 kg and would require only 50 kg extra feed.

The complete removal of fishmeal in one of this study’s diets resulted in the lowest FIFO value of 0.54 (or 540 kg wild caught fish per 1000 kg of fish produced). In the diets with only partial removal of fishmeal (i.e. low or high periphyton), calculated FIFO was 1.07 and 0.94, respectively; while the highest FIFO value of 1.36 was calculated for the control diet with 20% fishmeal and 10% fish oil.

Besides the present study using periphyton, other protein sources have been examined for their feasibility to replace fishmeal in diet of seabream including bacterial protein, plant or animal meals, or algae, as summarized in Table 4. Since data on the saving in feed costs is available only in one of these studies^15^, comparisons between the results could only be made regarding fish performance, the portion of fishmeal that was replaced, and the FIFO index which was calculated for the reported data by this study’s authors. Among the previous studies mentioned, only in a few of them was fishmeal totally removed from pellets: via use of alternative plant proteins^7^, vegetable meal^8^, macroalgae^15^, or the presently examined marine periphyton. In other trials, reduction in fishmeal content was at a rate of between 3 and 75% but resulted in at least 14% of fishmeal still included in the new experimental diet. It is also important to note that the length of the present trial was 4.5 months, which is much longer than the length of many of the other studies of 6–8 weeks reported here in Table 4. Thus, the reduction in growth observed in the diet with no fishmeal during the latter end of the experimental period can be attributed to the cumulative lack of this ingredient. The use of animal meal such as krill or meat and bone meals for fishmeal replacement was efficient in improving fish growth performance and FCR but none resulted in a FIFO index below 1. In fact, in the case of diets with krill meal, this index was even higher than in the original diet (1.3 vs. 1.2), since both fish- and krill-meal in the experimental diet were included in the calculation of the fish-in value, which was 25% as compared to only 20% in the control diet with fishmeal alone^52^. A FIFO index below 1, as was measured in the experimental diets tested in the current study (0.5–0.9), was obtained only in studies in which fishmeal was totally removed from diet, with the sole exception of one diet using a plant protein mixture^7^. The inclusion of green macroalgae *Ulva lactuca* in a fishmeal-free diet of seabream revealed lower SGR and less efficient FCR compared to the values measured in the current study where growth rate in fishmeal-free diet was 1.7% day^−1^ and required only 1.3 kg of feed for every kg of produced fish. A similar FCR was measured only in one other fishmeal-free experimental diet, using wheat gluten^7^.

**Table 4:** Comparison of current research with other trials on fishmeal replacement with alternative sources in diet of carnivorous sea bream.

Alternative protein source	FM content in control diet (%)	Change in FM content in new diet	Effects on sea bream growth performance	Fish oil content in new diet (%)	FIFO ratio of new diet (calculated)	References
Krill meal	20	FM reduction to 18.5, 17, or 16%	Improved SGR (1.3 vs. 1.2) and FCR (1.18 vs. 1.22) in diet with 16% FM and 9% krill meal	6	1.33	^52^
Alfalfa protein	70	FM reduction to 61% with 14% of alfalfa in feed	No significant change in FCR (~ 1.45)	4.6	3.46	^70^
Hydrolysed fish protein	22.25	FM reduction by 20%	No effect on FCR (~ 1.5)	6.9	1.34	^71^
Autolysed yeast	22.25	FM reduction by 20%	FCR slightly increased (to 1.6)	6.9	1.44	^71^
Soy protein concentrate or enzyme treated soy protein	70%	FM reduced to 30, 17, 16 or 15%. Soy protein or treated soy protein was used	FCR below 1.25 in all diets. In diet with 30% of FM and soy protein concentrate FCR = 1	12–14	1.5	^72^
Mixture of plant protein sources	70%	FM reduced to only 17.6% (75% reduction in FM)	FCR increased significantly in plants diet (1.3 to 2.3) but no effect was obtained in SGR	15	2.72	^9^
Hazelnut meal	63%	FM reduced by 10, 20, 30, or 40%	Improved SGR (1.45 to 1.6) and FCR (2.1 to 1.84) at 10% reduction in FM. No effect under other diets	8–10	4.1	^73^
Soy protein concentrate; wheat gluten; corn gluten	68	FM was totally removed and replaced by each of the plant meals	Only wheat gluten improved weight gain and reduced FCR (1.3 to 1.2)	18–20	0.85	^7^
Mixture of soy protein, wheat gluten, and corn gluten	68	FM was reduced by 25, 50, 70, or 100%	Improved weight gain and FCR (1.3 to 1.2) in diets with 50 or 75% less FM	14–18	1.43	^7^
Vegetable meal	59	FM was totally removed	Growth performance decreased in FM- free diet (SGR = 0.7; FCR = 2.4)	9	0.8	^8^
Meat and bone meal	57	FM was reduced by 50 or 75%	50% reduction in FM did not influence growth performance, SGR (2.5) and FCR (1.5)	9	1.97	^74^
Green macroalgae *Ulva pertusa*	50	FM was reduced by 9%	SGR was relatively similar (1) but FCR was higher with *Ulva* meal (2.3 vs. 2.1)	5	3.8	^75^
Green macroalgae *Ulva lactuca*	26	FM was reduced by 9, 23, or 100%	Full removal of FM did not influence growth performance, SGR (1.4) and FCR (1.7)	0.25	< 0.1	^15^
Marine periphyton	20	FM was reduced by 25, 50, or 100% removed	25 and 50% reduction in FM did not influence growth performance	10	0.5–0.9	Current study

All represented data were taken directly from the identified references, while FIFO ratio was calculated for the reported results using the recommended equation.

However, among all reported diets, the use of *Ulva lactuca* in seabream diet was by far the most successful in terms of the FIFO index value (< 0.1). This is particularly due to the low content of fish oil, only 0.25%, in that experimental diet^15^ which is significant as compared to all other examined diets. Although not addressed in the current study, a significant reduction in fish oil content may also be achieved with the use of marine periphyton, which contain high levels of essential fatty acids^46^. An interesting outcome from the present study was the relatively protein-rich (59%) but lipid-poor (23%) fish flesh when fed with the fishmeal-free diet with high periphyton content. As all diets contained the same level of crude protein and lipids (41 and 16.5%, respectively) it is assumed that such differences resulted from the origin of the protein source in the diet, i.e., the inclusion of periphyton in the feed pellets.

Despite this study’s promising results regarding the potential to support fish growth with periphyton-containing aquafeeds with the consequent benefit in both financial and environmental aspects, it did not consider some of the known drawbacks that have been correlated with the long-term use of plant diets in feeding of *S. aurata* such as the lower expression of inflammatory and immune related genes^53^ and a less diverse microbiota community in the fish gut^8^. However, in recent years, many of these drawbacks have been mitigated through supplementation with feed additives. Sodium butyrate has been suggested to have an opposite force to plant ingredients in diets of marine teleost through the prevention of growth retardation in parasitized fish and a positive effect on the gut microbiota by increasing its diversity^54^. Supplementing *S. aurata* with sodium butyrate was efficient in preventing inflammation of the intestinal epithelium and preserving the integrity of the intestinal barrier in *S. aurata* fed on plant-based diets low in fishmeal and fish oil^55^. Other alternatives included a medicinal leaf extract from sage (*Salvia officinalis*) and lemon verbena which improved the biological mechanisms associated with the immune system, gut integrity, and cellular proteolytic pathways in *S. aurata* when fed a plant- based diet^56^. Therefore, we can cautiously assume that such drawbacks, if occurring in *S. aurata* fed with periphyton-containing diets, may be mitigated through the use of similar feed additives.

The present results indicate a valuable and efficient dual use of marine periphyton for both mariculture wastewater treatment and fishmeal replacement in sea bream diets, while highlighting the great advantage of this raw material over alternative protein-rich raw materials examined due to its role in reducing aquaculture footprint through nutrient recapturing and recirculation and significant savings in fish production costs.

## Materials and methods

### Animals

The sea bream fish used in this study came from a stock maintained in the local aquaculture facility at NCM. Individuals were siblings from a single hatch.

### Ethics

This study was approved by the Agricultural Research Organization Committee for Ethics in Experimental Animal Use, and was carried out in compliance with the current laws governing biological research in Israel (Approval number: IL-600/15). The number of fishes that were sacrificed for further analysis was selected following the minimal criteria for statistical analysis. Sacrificing of fish was performed following anesthetizing with MS-222.

### Experimental diets

**Figure 1:**
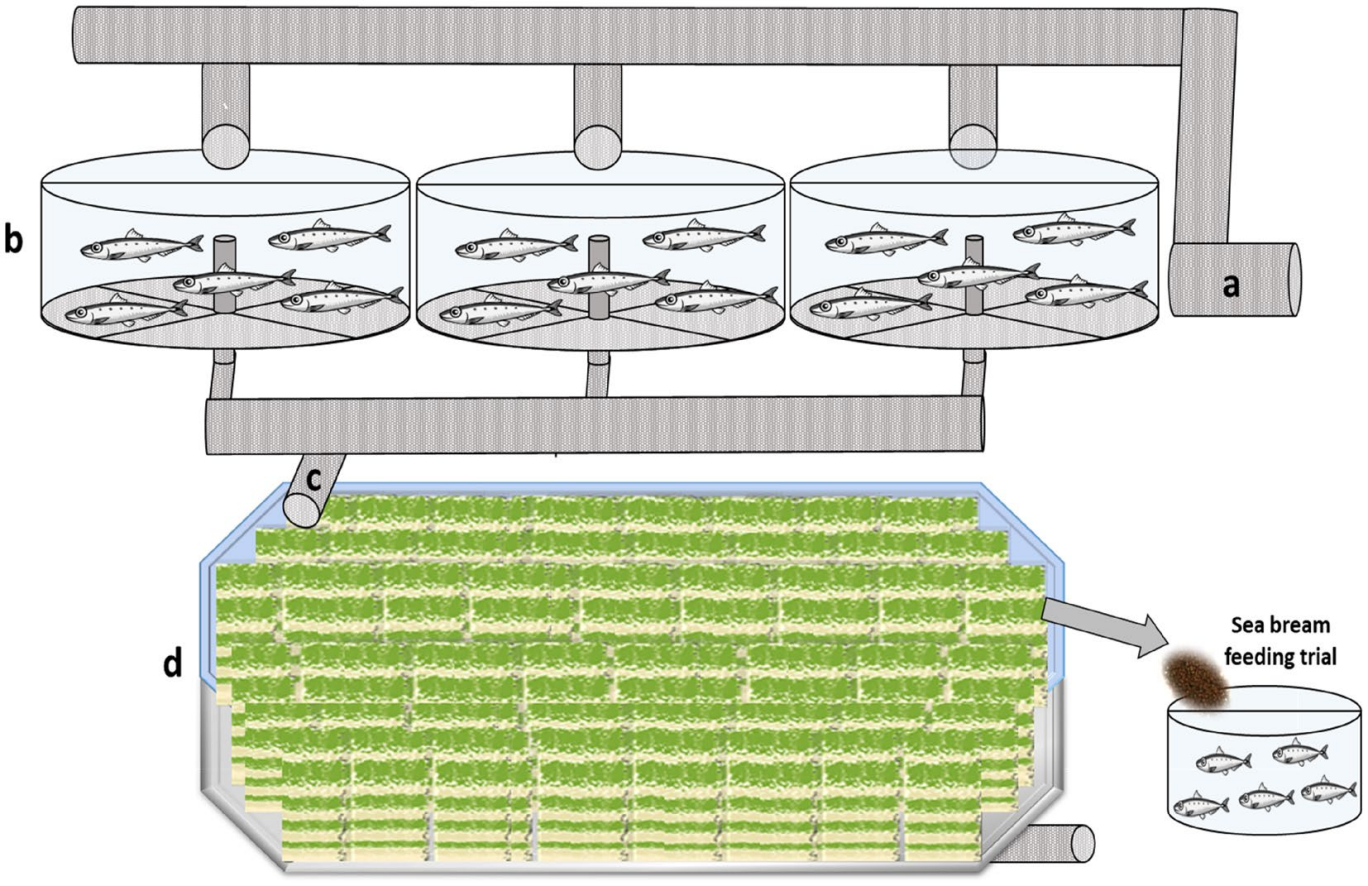
Schematic diagram (not to scale) of the periphyton production system for feeding trial. The IMTA system consists of three fish culture ponds (**b**) supplied with fresh seawater from the Gulf of Aqaba (**a**). Fishpond effluents (**c**) are transferred to the periphyton biofilter (**d**) and the produced biomass (after drying) serves for preparation of the experimental diets for the fish feeding trial.

Four isoproteic (41.7 ± 0.4% crude protein or CP) and isolipid (16.7 ± 0.3% crude lipid or CL) diets were formulated to examine the effect of fishmeal replacement with periphyton. The ingredients in the different diets are summarized in Table 5. Briefly, the control diet was a commercial aquafeed for sea bream (2.4 mm, Ra'anan, Israel) containing 200 g fishmeal kg^−1^. This ingredient was reduced to 150, 100, and 0 in the experimental diets by adding amounts of periphyton meal in pellets that would maintain CP level in the aquafeeds at 41.6% (± 0.4) (Table 5). Prior to inoculation in formulated feed, periphyton was grown in the biofilter of the local IMTA system at the National Center for Mariculture (NCM) in Eilat (Fig. 1).

**Table 5:** Feed ingredients and biochemical content in experimental diets with periphyton and reduced fishmeal as compared to control commercial diet; and the biochemical content in marine periphyton from biofilter of the IMTA system.

	Marine periphyton	Control	Low Periphyton	High periphyton	High periphyton–no fishmeal
Fishmeal		200	150	100	–
Periphyton (27% protein)		0	125	250	250
Poultry meal		120	120	120	120
Wheat flour		240	120	30	30
Wheat gluten		60	60	60	60
Soybean protein		80	80	80	80
Corn gluten		90	90	90	90
Soybean meal		90	135	150	250
Fish oil		100	100	100	100
Choline chloride		5	5	5	5
Vitamins and minerals mix		5	5	5	5
Lysine		5	5	5	5
Methionine		3	3	3	3
Vitamin C		2	2	2	2
Protein	28	40.7	42.3	42.3	41.3
Lipids	5	17.5	16.5	16.2	16.7
Carbohydrates	30	34.2	29.9	26.4	25.9
Ash	37	7.6	11.3	15	16.1

Ingredients content in feed is provided in g kg^−1^ feed. Protein, lipids, carbohydrates, and ash content are in % of dry biomass (periphyton or pelleted feed).

The fish culture system consisted of three 40 m^3^ fishponds for production of grey mullet (*Mugil cephalus*) in which fish were cultured at a stocking density between 10 and 15 kg/m^3^ while fed a recommended commercial aquafeed (Ra'anan, Israel) at a rate of 2.0% body weight day^−1^. The periphyton biofilter in this system was an octagonal pool with an effective working volume of 100 m^3^, alimented with the nutrient-rich fishpond effluent. The periphyton in this biofilter developed spontaneously on an artificial substrate of white plastic nets (mesh size 2.5 × 2.5 mm) following previous recommendations^29,30^. In order to culture the required biomass of periphyton within a single batch, the total substrate area in the biofilter was set at 215 m^2^, considering both sides of the net as effective surfaces for biomass development. Nets were suspended from wires at the top of the tank and maintained in a vertical position in the water column using stainless steel weights. After 2 weeks, all the nets were removed from the pond, gently rinsed with freshwater, and left outdoors for air drying for 36 h. The attached dry periphyton was then removed from the nets using a toothbrush, weighed, ground into 2.4 mm size particles and stored at 4 °C until feed preparation. Samples of periphyton were used to determine levels of protein, lipids, carbohydrates, and ash in the dried biomass (Table 5) in order to prepare the experimental diets with periphyton as per these biochemical content results. The relatively high ash content in the periphyton, as well as in all feeds with a high level of periphyton, may be attributed to the relatively high diatom content in the periphyton biomass at this time of the year, based on our results from earlier studies on the community composition of marine periphyton and other studies conducted during the same time of the year^29,30^.

### Experimental design

A total of 360 fingerlings of *S. aurata* from a single hatch were transferred from the local nursery at NCM and distributed equally into twelve cylindrical polyethylene tanks of 380 L each with a conical bottom (30 fish per tank). Tanks were supplied with filtered Red Sea water (40 ± 0.5 ppt salinity) pumped from an inlet located 300 m offshore at a depth of 20 m. The water entered each tank at the top, perpendicular to the tank wall, at a constant flow rate of 540 L h^−1^, and exited from a central outlet at the bottom through an external vertical stand-pipe. Each tank was covered with a white net top to allow light in, while preventing access to animals, as the tanks were located in an open greenhouse. Air was supplied using air-stones. Oxygen level was measured daily and maintained steady at 5.9 ± 0.2 mg/l during the entire experiment. Temperature and pH were measured twice a week and maintained steady at 26.6 ± 0.9 °C, and 8.3 ± 0.1, respectively. Fingerlings were allowed to acclimate to the experimental tanks for 13 days, during which all were fed with the commercial aquafeed of the control diet at a rate of 2% of body weight (BW). After this period, weight and body length of each individual fish was recorded and fish were assigned to the new diets. Each diet was examined in triplicate tanks over a period of 132 days. Feed was provided manually three times a day (08:00, 12:00, and 16:00). The amount of feed was calculated according to periodic weighing events (every 2–3 weeks) using the recommended feed intake equation by Lupatsch and kissil^57^ which considers fish weight and weight gain, as well as the ambient temperature of water in the culture tank. All weighing events were performed after fish sedation with clove oil.

### Calculations of fish growth performance

Fish specific growth rate (SGR), yield, survival, ratio between gut and body length (RGL) and Zihler's index of the ratio between the intestine length and fish body weight (ILW), food conversion ratio (FCR), protein efficiency ratio (PER) and protein productive value (PPV), were all calculated at the end of the experiment based on measurements and the documented data.

Growth yield was calculated by the difference between initial and final weights and the specific growth rate (SGR) was calculated as:

$${\text{SGR}} = 100 \times \left[ {\ln \left( {W_{T} /W_{0} } \right)} \right]{/}t$$

where W_0_ = initial biomass, W_T_ = final biomass, and t = the culture period in days. RGL was calculated following the ratio between gut:body length (both in cm).

ILW Zihler's index was calculated following the original equation and later recommendations for studies on marine fish^58,59^.

Food conversion ratio (FCR) was calculated as:

$${\text{FCR}} = {\text{Fg/}}\left( {{\text{W}}_{{\text{t}}} - {\text{W}}_{0} } \right)$$

where Fg = feed administered (g); and W_0_ and W_t_ are the initial and final mean wet weight (in g), respectively.

Protein efficiency ratio (PER) was calculated as: PER = wet weight gain (g)/crude protein consumed (g). Protein productive value (PPV) was calculated as: PPV = protein gain (g)/crude protein consumed (g) × 100.

### Analytical methods

Measurements of the biochemical composition in periphyton, pelleted diet, and fish included levels of protein, lipids, carbohydrates and ash. Fish analyses were performed on five fish that were sacrificed from the stock school at the beginning of the experiment and on three fish from each of the culture tanks at the end of the experiment. Protein content was determined following the Kjeldahl method for total nitrogen measurement and recommended N to protein conversion factor^60^. The lipid content was measured after chloroform–methanol extraction^61^, and carbohydrate level was determined using the phenol sulfuric acid method^62^. Dry matter was measured following weighing prior to and after drying at 105 °C for 24 h and ash was determined after further incineration for 24 h at 550 °C in a muffle furnace^63^.

### Statistical analyses

All data analyses were performed using R software (R version 4.0.3) while setting the significant threshold at *P* < 0.05. A linear mixed model (LMM) was created using the R package ‘lme4’^64^ to evaluate the effect of diet, time and their interaction on fish performance. Coefficient of determination (R^2^) was calculated using the ‘r2glmm’ package^65^, and Tukey’s post-hoc comparison of the means was obtained using the package ‘emmeans’^66^. Normal distribution of the data was verified using the Shapiro–Wilk normality test and homoscedasticity with the Bartlett test^67^. In cases where the homoscedasticity required for a simple one-way ANOVA test was not met, Welch one-way test (one-way ANOVA test with no assumption of equal variances) was performed.

### Fish in–Fish out and economic analyses

Calculation of the fish in–fish out ratio (FIFO) considered the two fish-derived ingredients in the formulated feeds: fishmeal and fish oil. FIFO was calculated following the equation recommended by Jackson and Aldon^68^ and Shepherd and Jackson^50^:

$${\text{FIFO}} = {\text{FCR }} \times \, \frac{{\left( {{\text{Fishmeal level }} + {\text{ fish oil level in diet}}} \right)}}{{\left( {{\text{fish meal yield }} + {\text{ fish oil yield from wild caught fish}}} \right)}}.$$

Economic analyses included the calculation of the price of each of the formulated feeds ($/kg feed, in US dollars) according to costs of the various ingredients on the date of preparation as received from suppliers or in the Index Mundi database^69^ and their content in the formulated diet. Savings in feed costs were calculated against the calculated cost of the control diet. The savings in feed costs were also analysed after normalization of feed cost to the obtained data. For each diet, the total cost of producing 1 kg fish was calculated according to feed cost and the measured FCR.

### Ethics and approval of animal experiment

The study is reported in accordance with the Animal Research Report of in vivo Experiments (ARRIVE) guidelines. The research was performed following all relevant ethics. This study was approved by the Agricultural Research Organization Committee for Ethics in Experimental Animal Use, and was carried out in compliance with the current laws governing biological research in Israel (Approval Number: IL-600/15).
