# Author Correction: Fishmeal replacement by periphyton reduces the fsh in fsh out ratio and alimentation cost in gilthead sea bream *Sparus aurata*

**DOI:** 10.1038/s41598-022-11695-7

**Published:** 2022-05-03

**Authors:** Gilda Savonitto, Roy Barkan, Sheenan Harpaz, Amir Neori, Helena Chernova, Antonio Terlizzi, Lior Guttman

**Affiliations:** 1 Israel Oceanographic and Limnological Research, The National Center for Mariculture, 8811201 Eilat, Israel; 2 Department of Life Sciences, University of Trieste, Trieste, Italy; 3 Department of Life Sciences, Ben-Gurion University of the Negev Eilat Campus, 84105 Beer-Sheva, Israel; 4 Department of Poultry and Aquaculture, Institute of Animal Science, Agricultural Research Organization, Rishon LeZion, Israel; 5 Morris Kahn Marine Research Station, Marine Biology Department, The Leon H. Charney School of Marine Sciences, University of Haifa, 3498838 Haifa, Israel; 6 The Interuniversity Institute for Marine Sciences in Eilat, 8810302 Eilat, Israel; 7 Stazione Zoologica Anton Dohrn, Napoli, Italy

Correction to: *Scientific Reports* https://doi.org/10.1038/s41598-021-00466-5, published online 25 October 2021

The original version of this Article omitted an affiliation for Amir Neori. The correct affiliations are listed below.

Morris Kahn Marine Research Station, Marine Biology Department, The Leon H. Charney School of Marine Sciences, University of Haifa, 3498838 Haifa, Israel

The Interuniversity Institute for Marine Sciences in Eilat, 8810302, Eilat, Israel

The original Article has been corrected.

